# Water-soluble substituted chitosan derivatives as technology platform for inhalation delivery of siRNA

**DOI:** 10.1080/10717544.2018.1440668

**Published:** 2018-03-01

**Authors:** Victoria Capel, Driton Vllasaliu, Peter Watts, Philip A. Clarke, Dominic Luxton, Anna M. Grabowska, Giuseppe Mantovani, Snjezana Stolnik

**Affiliations:** a Division of Molecular Therapeutics and Formulation, School of Pharmacy, University of Nottingham, University Park, Nottingham, United Kingdom;; b Archimedes Development Limited, Albert Einstein Centre, Nottingham Science and Technology Park, University Boulevard, Nottingham, United Kingdom;; c Division of Cancer and Stem Cells, School of Medicine, University of Nottingham, Nottingham, United Kingdom

**Keywords:** Chitosan, siRNA delivery, siRNA complex, lung inhalation, IVIS imaging

## Abstract

Despite research efforts full potential of siRNA-based therapeutics has not yet been fully realized due to a need for suitable, effective delivery formulations. Here, we examine a potential of a new class of water-soluble chitosans as siRNA platform for pulmonary delivery. The system is based on piperazine-substituted chitosans, a material designed to integrate established, safe application of chitosan for mucosal administration with novel properties: the piperazine-substituted chitosans are freely water-soluble at physiological pH, possess low cytotoxicity (no significant reduction in cell viability up to 0.1 mg/ml), and provide efficient incorporation of siRNA into sub-300 nm colloidal complexes (at relatively low polymer/siRNA ratio of 5:1). *In vitro*, the complexes achieved silencing of a model gene at a level of 40–80%, when tested in a panel of lung epithelial cells. Considering the formulation ‘developability’, there were no significant changes in the complexes’ size and integrity on aerosolisation by microsprayer (PenCentury™) device. Following intratracheal aerolisation, the complexes deposited throughout the lung, although relatively inhomogeneously, as judged from IVIS imaging of the isolated mouse lung (visualizing DY647-siRNA). *In vivo* data illustrate absence of adverse effects on repeated administration of complexes and significant tumor reduction in atopical lung cancer model in mice. Altogether, the data illustrates potential of substituted chitosan derivatives to be utilized as a safe system for inhalation delivery of siRNA.

## Introduction

RNA interference is a natural phenomenon mediated by short 21–23 bp RNAs (siRNA), which is being exploited to selectively silence genes involved in disease. This rapidly expanding area has led to studies with therapeutics siRNA for a range of diseases, including HIV, respiratory viruses, and cancer. Effective delivery, however, remains the most significant barrier to realizing the full potential of siRNA silencing in clinic. Cationic polymers have been used to incorporate siRNA via electrostatic interactions into nano-sized complexes; including studies employing chitosan. Chitosan is ‘generally considered as safe’ (GRAS) pharmaceutical excipient, and has previously been studied for siRNA delivery to the lung; chitosan-siRNA nanoparticles (formulated at N:P ratio of 23:1) were administered intranasally to EGFP-expressing transgenic mice and resulted in a 43% reduction of EGFP expression in the bronchioles, compared to the untreated control (Howard et al., 2006). However, a limitation to formulation developability of chitosan is its poor solubility at physiological pH (Katas & Alpar, 2006). To overcome this problem, chitosan derivatives have been developed by, for example, modifications of either free primary amine groups to create quaternary ammonium products of chitosan, such as trimethyl chitosan (TMC), or chain PEGylation (Casettari et al., 2010), with both approaches increasing the product’s water solubility. Here, we investigated a family of piperazine-substituted chitosans, a material aimed to integrate established application of chitosan for mucosal administration (Casettari et al., 2010), with improved developability as siRNA delivery agent, based on their water solubility at physiological pH.

We tested the potential of piperazine-chitosans in siRNA silensing of EGFR, a pro-angiogenic factor up-regulated in non-small cell lung cancer (NSCLC), one of the most malignant forms of lung cancer. EGFR provides an essential survival signal to tumor cells through the activation of the expression of anti-apoptotic factors such as Akt and protein kinase C (Shen & Kramer, 2004). Unfortunately, therapies such as anti-angiogenesis monoclonal antibodies or tyrosine kinase inhibitors have failed to show significant advantages over chemotherapy and have failed to improve long-term patient survival (Martinelli et al., 2009). The siRNA therapy to silence EGFR offers a potential alternative, however, ‘naked’ siRNA delivery to the lung has not proven efficient (Moschos et al., 2007). In the presenct work, we appreciated a need for a delivery system to protect siRNA from a relatively high levels of RNase activity in the lung (ThermoFisher Scientific, 2017), as well as poor cellular internalization. Water soluble substituted chitosan-siRNA complexes were formulated with the product attributes appropriate for the development of an inhalation formulation, while low N:P ratio (5:1) was aimed at reducing the lung burden of an excess of siRNA condensing material.

## Materials and methods

Water-soluble piperazine chitosan derivatives were provided by Archimedes Pharma (Nottingham, UK). Water-soluble piperazine chitosan derivatives were provided for this study by Archimedes Pharma (UK). These were supplied, synthesized, and characterized, as described previously, by (Holappa et al., 2006). The structural characterization and physicochemical properties of modified chitosans used in the study are summarized in Figure S1 and Table 1. GAPDH-specific siRNA was purchased from Dharmacon (CO, USA). Lipofectamine 2000 was purchased from Invitrogen (Paisley, UK) and used as *per* supplier’s instructions. All other chemicals, unless otherwise stated, were obtained from Sigma-Aldrich (Poole, UK). H1299, A549, and Calu-3 cell lines were purchased from the American Type Culture Collection (ATCC, Manassas, VA, USA). H1299 cells were routinely cultured in RPMI-1640 medium supplemented with 10% *v/v* fetal bovine serum, 1% *v/v* penicillin/streptomycin, and 1% *v/v*l-glutamine at 37 °C/5% CO2. A549 and Calu-3 cells were cultured in Dulbecco’s Modified Eagle’s Medium (DMEM) and Eagle’s minimal essential medium (EMEM, Sigma-Aldrich, Dorset, UK) respectively, supplemented as previous. EMEM was further supplemented with sodium pyruvate (1% *v/v*). GAPDH targeting siRNA (5′-UGGUUUACAUGUUCCAAUAUU-3′ (sense), 3’UUACCAAAUGUACAAGGUUAU P-5’ (antisense)) was purchased from Dharmacon (UK).

**Table 1.** Structures and properties of piperazine chitosans used in the study.

Piperazine substituent of chitosan	Polymername	Degree ofsubstitution (%)	Mw (kDa)	Mn (kDa)	Mw/Mn	Averagemonomer charge*1	Calculated 1:1charge ratio*2
	NQ30	30	42.9	24.7	1.73	0.33	3.03
	NQ60	60	35.0	16.6	2.09	0.60	1.67
	MQ4-42	42	34.5	17.4	1.98	0.46	2.16
	MQ4-87	87	28.6	17.2	1.66	0.87	1.15
	MQ1-46	46	39.2	20.4	1.92	0.50	2.00
	MQ1-85	85	34.2	21.8	1.57	0.85	1.18
	DQ39	39	24.0	39.5	1.65	0.83	1.21
	DQ56	56	33.5	32.1	1.04	1.15	0.87

All substituted chitosans have degree of deacetylation of 85%. NQ: non-quaternarised, MQ1: mono-quaternary, center on the 1-position of piperazine ring; MQ4: mono-quaternary, center on the 4-position of piperazine ring; DQ: di-quaternary substitution. Numeric value in the name refers to the percentage piperazine substitution.

*1 Estimated from polymer composition

*2 Calculated assuming protonation in pH 7.4 buffer as discussed in the text.

Silencer Cy3^TM^ and DY647^TM^ labeled siRNA targeting GAPDH was purchased from Thermo Fischer Scientific. EGFR (5′-CACAGUGGAGCGAAUUCCU-3′) ONTARGETplus *in vivo* siRNA and negative control siRNA were purchased from Dharmacon (UK).

### Formation of substituted-chitosan – siRNA complexes

Complexes were prepared in Tris-HCl buffer (10 mM, pH 7.4) by adding equal volumes of polymer to siRNA solutions to give the calculated monomer:nucleotide ratio:

$$Mass\;polymer=\frac{Mw\;polymer\;RU}{Mw\;siRNA\;RU}\times \;ratio\;\times \;mass\;siRNA$$

where ‘RU’ refers to the average molecular weight of the repeating unit of the polymer or siRNA. It should be noted that in a subsequent text substituted-chitosan to siRNA ratios are not defined, as generally used, charge per charge ratio (N:P), but rather as monomer per siRNA nucleotide ratio (M:N). This is due to the fact that protonation of both unsubstituted chitosan ‘backbone’ monomers, and substituted piperazine ring, is pH dependent, meaning that the polymers’ charge density will be dependent on their environment.

### Ethidium bromide displacement assay

Ethidium bromide (EtBr) (4 µl, 1 mg/ml) was added to a cuvette containing 10 mM Tris-HCl (1 ml) and fluorescence measured (Hitachi F-4500 fluorescence spectrophotometer) at λ_ex_560 and λ_em_ 605 nm. siRNA solution (10 µg) was then added and fluorescence recorded. Polymer solutions (1 mg/ml) were added into the cuvette in a stepwise manner to give the required monomer:nucleotide ratio. After each polymer addition, samples were gently agitated, followed by fluorescence measurements for 2–3 minutes. Sample measurements were carried out in triplicates. The fluorescence for each ratio was calculated relative to the maximum fluorescence for the EtBr-siRNA solution and corrected for the background fluorescence of EtBr using the following equation:

$$Relative\;fluorescence\;\left(\%\right)=\frac{{ƒ}_{polyplex}-{ƒ}_{EtBr}}{{ƒ}_{siRNA}-\;{ƒ}_{EtBr}}\times 100$$

where ‘ƒ_EtBr_’ is the fluorescence of EtBr in buffer, ‘ƒ_siRNA_’ is the fluorescence of EtBr plus siRNA in buffer, and ‘ƒ_complex_’ is the fluorescence of EtBr plus complex in buffer:

### Agarose gel electrophoresis

Aliquots of polymer solution were added to the siRNA solution (1 μg). Complexes were prepared as described above over the following range of monomer:nucleotide molar ratios: 0.25:1, 0.5:1, 0.75:1, 1:1, 1.25:1, 1.5:1, 1.75:1, 2:1, and 5:1. Free polymer and free siRNA served as controls. The samples were briefly mixed by vortexing and incubated at room temperature for 30 min. Loading buffer (2 μl) was added to the samples and loaded into 1.2% agarose gel containing EtBr (1 μg/ml). Electrophoresis was carried out at 80 V in 1X Tris-acetate-EDTA buffer (pH 7.4) for 1 hr. The siRNA bands were visualized under UV transillumination. Polymer bands were stained by immersing the gel in staining solution (10/50/40 glacial acetic acid, methanol, double distilled water, 1% *w/v* Coomassie blue) for 1 hour, followed by washing with destaining solution (10/10/80 glacial acetic acid, methanol, double distilled water) overnight.

### Particle size analysis

The mean hydrodynamic diameter and particle size distribution of polymer-siRNA complexes was determined by dynamic light scattering (DLS) using a Viscotek 802 system (Malvern Instruments , UK). Complexes were suspended in 10 mM Tris-HCl buffer (pH 7.4). The results are expressed as the mean of three separate measurements, with each measurement showing a mean value of 10 runs. All measurements were carried out at 25 °C.

### Release of siRNA from complexes

Agarose gels were prepared at a concentration of 1.2% *w/v* in 1X TBE buffer containing 0.5 μg/ml ethidium bromide. Polyplexes (20 μl) containing 1 μg of siRNA at a 5:1 ratio were formed over 24 hours. l-glutathione 2.5 mM (9.22 μL, 5 mg/ml solution) was added to the polyplexes formed with thiolated polymer, and they were incubated at 37 °c for 30 minutes. Following this, heparin was added to certain polyplexes at concentrations of 0.1 U heparin/μg siRNA (6 μL, 0.5 mg/ml) and 0.2 U heparin/μg siRNA (6 μL, 0.25 mg/ml), followed by gel electrophoresis assay.

### Cell toxicity assays

The effects of piperazine chitosans on cellular metabolic activity (indicator of cell viability) was determined by an MTS assay. H1299 cells were cultured on 96-well plates (10,000 cells/well) for 24 hours. The culture medium was replaced with serum-free medium containing polymer solutions (0.0001–1 mg/ml) and cells were incubated with the samples for 4 hours. Samples were removed and the MTS assay was carried out according to supplier’s instructions. Results are given as mean values of eight repeats (± SD). IC_50_ values were calculated using GraphPad Prism by non-linear regression.

Toxicity of DQ39 polymer was futher tested in H1299, A549, and Calu-3 cells. Cells were cultured on 96-well plates (10^4^ cells/well) for 24 hours prior to the toxicity study. Culture medium was then replaced with serum-free equivalents (RPMI-1640, DMEM or EMEM, respectively) containing polymer solutions (0.0001–1 mg/ml) and incubated for cells were incubated with the samples for 4 hours. Samples were removed and MTS assay conducted according to supplier’s instructions. Relative cell viability was calculated via comparison with cells treated with culture medium or 0.2% *v/v* Triton-X (as negative or positive controls, respectively). Results are given as mean values (*n* = 8). Half maximal inhibitory concentration (IC_50_) values were calculated with GraphPad Prism by non-linear regression.

### *In vitro* silencing of a model gene

H1299 cells were seeded on 24-well plates (10^5^ cells/well) and cultured to ∼70% confluence. siRNA-substituted chitosan complexes (corresponding 100 nM siRNA) were added to the cells in serum-free medium (HBSS:HEPES pH 7.4) and incubated for up to 4 hours. Samples were then removed and replaced with fresh culture medium and cells cultured for 44 hours. GAPDH activity was analyzed using the KDalert GAPDH kit (Ambion, USA), following the supplier’s protocol, as a commonly used knock-down assay. Relative GAPDH activity was calculated against untreated cells.

### Droplet size and spray pattern analysis

Droplet-size analysis of aerosols was conducted by laser diffraction using a Malvern Spraytec® (Malvern, UK) with RT Sizer software. Actuation of the microsprayer was conducted manually, at a distance of 4 cm from the laser beam. All measurements were made at room temperature (20°–23 °C). The focal length of the lens used was 100 mm, with a corresponding droplet-size range of 0.5–200 μm. The refractive index and absorption coefficient settings were a media refractive index of 1.00 + 0.00i (air) and particulate refractive index of 1.33 + 0.00i (water). Data is reported as volume diameter defined by 10%, 50% (volume median), and 90% of the cumulative volume undersize; Dv10, Dv50, and Dv90, respectively.

The spray pattern from the microsprayer was visualized by spraying formulations containing a dye onto TLC paper. The minimum and maximum diameters of the spray pattern were measured by digital calipers. The spray pattern was characterized by determining the ‘effective diameter’ and ovality. ‘Effective diameter’ was defined as the averages of the maximum and minimum diameters of three sprays and the ovality was expressed as the maximum diameter divided by the minimum diameter.

### *In vitro* toxicity to A549-luc cells

The effects of EGFR targeted siRNA on the cell toxicity to A549 luciferase expressing cells (A549-luc) *in vitro* was measured by MTS viability assay. A549-luc cells were seeded on 96-well plates at 10^4^ cells/well and cultured for 24 hours. DQ39-siRNA complexes (formulated at a 5:1 M:N ratio) containing EGFR targeted siRNA, or negative control siRNA, were added to cells in serum-free culture medium 100 nM siRNA/well. After 4 hours, the complexes were removed and replaced with fresh serum-containing medium. Cells were incubated for a further 24, 48, or 72 hours, after which cell survival was measured by an MTS assay.

In these experiments, doxorubicin was employed for comparison. Doxorubicin was added to the cells (cultured as above) at 5 µM and cells were incubated for 24, 48, or 72 hours. After each time point, the samples were removed and an MTS assay carried out, as described above.

### *In vivo* studies

Animal studies were conducted under the UK Home Office Licence number PPL 40/3559, 19 b (3). NCRI guidelines for the welfare and use of animals in cancer research, and LASA good practice guidelines and FELASA working group on pain and distress guidelines were followed. Male MF-1 nude mice at six to seven weeks of age were obtained from Charles River, Harlow, UK. Mice were maintained in individually ventilated cages (Tecniplast, UK) within a barriered unit illuminated by fluorescent lights set to give a 12 hour light-dark cycle as recommended in the United Kingdom Home Office Animals (Scientific Procedures) Act 1986. The room was maintained at an air temperature range of 21 ± 2 °C and a humidity of 55% + 10%. Mice were housed in social groups during the procedure with irradiated bedding and provided with autoclaved nesting materials and environmental enrichment. Mice were terminated by an approved S1 method.

A549-*luc* tumor cells were suspended in standard formulation of Matrigel at 2 × 10^6^ cells per 100 μl. Tumors were established in MF-1 nude male mice by implanting subcutaneously into the left flank of each mouse. Tumor establishment and growth was monitored during the experiment by caliper measurements twice weekly, 2 D and 3 D optical imaging carried out under anesthesia in an IVIS Spectrum weekly, and at termination, to provide an optical post mortem. Tumor measurements and 3 D image reconstruction were made using Living Image (4.3.1 build) software (Caliper Life Sciences Hopkinton, MA, USA). To quantify the fluorescent intensity, a fluorescent calibrated unit (termed radiant efficiency), representing the fractional ratio of emitted photons per incident excitation photon, was used to compensate for non-uniform excitation light pattern.

Dosing was initiated in established tumors, those whose volume was demonstrably larger than the original implantation volume. Treatment groups (*n* = 8 mice per group) were as follows: (i) EGFR-siRNA-DQ39 complexes (10 µg siRNA, intratumoral injection, twice weekly) and (ii) negative control siRNA-DQ39 complexes (10 µg siRNA, intratumoral injection, twice weekly). All animals were sacrificed at the same time when tumor volume reached the maximum permissible size of 1.5 cm diameter in at least one mouse. Tumors were dissected and preserved for immunohistochemical analysis.

### Lung distribution

Male MF-1 nude mice, six to seven weeks old, were dosed intratracheally by the Microsprayer Aerosolizer (Model IA-IC, PennCentury, Philadelphia, PA, USA). Mice were anesthetized with ketamine/medatomidine (Ket 0.9 ml/Met 0.3 ml/NaCl 8.8 ml) dosed at 10 µl/g. Mice were restrained on the support rig and the instillate administered intratracheally using a Microsprayer Aerosoliser, guided by a pediatric otoscope. Each dose contained 10 µg of siRNA in 50 µl. Imaging was carried out in the IVIS Spectrum with parameters chosen for DY647 and the closest red-shifted filter set. A ‘blue light’ control filter set was also used to provide auto-fluoresence data for un-mixing and image analysis. For the lung IVIS analysis, mice were culled by an approved S1 method post initial imaging, while still under anesthetic; lungs were dissected and imaged as described above.

### Immunohistochemical analysis

Tumor xenograft sections (∼4 µm) were dewaxed in two sequential xylene washing, rinsed in three sequential methanol baths, and rehydrated in running tap water. Staining for EGFR was carried out using a modified Animal Research Kit to prevent protein on protein interactions according to manufacturer’s instructions (ARK, Dakocytomation, Agilent, Santa Clara, CA, USA): antigen retrieval was carried out by incubation with proteinase K, 0.05 mol/L for 10 minutes (Dakocytomation). Endogenous peroxidase was blocked with hydrogen peroxide, and tissue sections were incubated with either biotinylated mouse monoclonal anti-human EGFR (clone E30, 16.6 µg/ml, Dakocytomation) or biotinylated mouse IgG control serum (Dakocytomation) at matched concentrations for 60 minutes. Each primary treatment was pre-blocked with mouse protein blocking agent to the appropriate concentration. Visualisation was via streptavidin-HRP secondary antibody for 15 minutes, followed by liquid system DAB chromogen for 5 minutes. Sections were counterstained with Mayer’s Haemalum and then dehydrated and cleared through sequential methanol and xylene rinses and cover-slipped with DPX adhesive (Sigma).

Analysis of staining was carried out by the H-score method, which takes into account the percentage of tissue stained and its staining intensity. Staining levels (percentage) were divided into high, medium, and low intensity for each region of interest and a multiplication factor (weighting) was applied to each: 3 for high intensity, 2 for medium intensity, and 1 for low intensity. These data were added to give the H-Score value, which at its maximum will not exceed 300.

### Statistical analysis

GraphPad Prism 7 software (La Jolla, CA, USA) was used for data analysis. Comparisons between two groups were made using independent samples Student’s *t*-test and one way analysis of variance (ANOVA) was applied for the comparison of three or more groups. Tukey’s multiple comparison test (*post-hoc*) was used in conjunction with ANOVA to evaluate differences in individual means in EGFR staining data. For other experiments, Bonferroni *post-hoc* test was applied for comparison of group means of three or more groups. *p* Values of < .05 were considered statistically significant. *, **, ***, and **** indicate *p* < .05, *p* < .01, *p* < .001, and *p* < .0001, respectively.

## Results and discussion

In the present work, we assessed potentials of a new class of water soluble, piperazine-substituted derivatives of chitosan, as condensing agents for development of inhalable siRNA delivery formulation. The piperazine derivatives were synthesized and characterized as previously described (Holappa et al., 2006) and summarized in Table 1. The derivatives vary with respect to the structure of the piperazine substituent, as well as the degree of substitution. These can be grouped into four categories depending on the nature of the piperazine substituent and location of the quaternary center as: non-quaternarized (**‘**NQ’), mono-quaternary with center on the 1-position of piperazine ring (‘MQ1’), mono-quaternary with center on the 4-position of piperazine ring (‘MQ4’) and di-quaternary (‘DQ’). In each of the four categories, derivatives with a high and a low degree of piperazine substitution were synthesized to investigate the effect of degree of substitution (in effect corresponding to a charge density), in addition to the effects of structure of the substituent. The polymers were designed to have similar molecular weights in order to remove this variable from the study. It should be noted that all substituted chitosan derivatives synthesized are water-soluble materials at physiological pH, a clear advantage over ‘un-modified’ chitosan from a product developability perspective.

**Figure 1.**
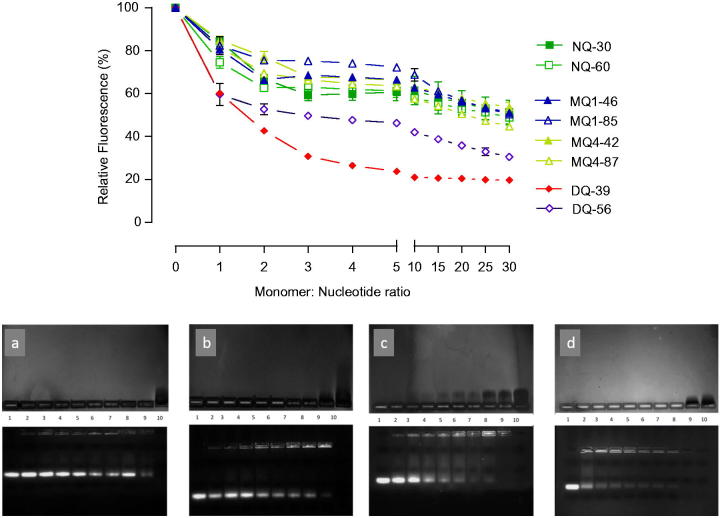
siRNA complexation by piperazine substituted chitosans. (A) EtBr displacement assays of piperazine substituted chitosans with siRNA. Polymers and EtBr containing siRNA solutions in 10 mM Tris-HCl buffer pH 7.4 mixed at specified at monomeric unit: nucleotide ratio; fluorescence level expressed as a% of the initial relative fluorescence of the EtBr siRNA complex (*n* = 3). (B) Agarose gel electrophoresis. The upper image shows the migration of free polymer, and the lower image shows the migration of free siRNA. The bands were visualized as described in the Methods section. Lane 1: siRNA control; lanes 2–9: polymer:siRNA complexes at ratios 0.25, 0.5, 0.75, 1.0, 1.25, 1.5, 2.0, and 5.0 to 1; lane 10: polymer control. Polymers: gel a shows MQ1-46; b: MQ4-42; c: NQ30; and d: DQ39.

Regarding the polymers’ ability to complex siRNA, an initial screening applying EtBr displacement assay (Figure 1(A)) illustrates that a plateau in siRNA complexation was achieved at relatively low monomer:nucleotide ratios of approximately 5:1, and that variations in the complexation ability are related to the piperazine-chitosan structural properties. For non-quaternary and mono-quaternary derivatives, regardless of the quaternary center position and degree of substitution, there is generally a lower level of EtBr fluorescence reduction, relative to di-quaternary piperazine chitosans, suggesting a higher siRNA binding affinity by the molecular structure of the latter derivatives. A similar trend is seen in electrophoretic migration gel assay (Figure 1(B)) where di-quaternary DQ39 derivative exhibited high-binding affinity for siRNA, as judged from disappearance of free siRNA in the gel at relatively low monomer:nucleotide ratios (∼0.5:1). However, the presence of a relatively intense EtBr fluorescence in the application wells for DQ39 complexes, even at monomer:nucleotide ratios of ∼5:1, would indicate the formation of ‘loose’ complexes, which allow EtBr intercalation with siRNA. The reasons for this may include an importance of ‘matching’ charge densities in polyelectrolyte interactions; two polyelectrolytes with ‘matching’ charge densities can form compact complexes while the charge density ‘mismatching’ results in the ‘swollen’ complexes (Hoogeveen et al., 1996; Danielsen et al., 2005) (Dautzenberg & Jaeger, 2002) hence making EtBr intercalation possible. The highly substituted di-quaternary piperazine-chitosan, DQ56, did not increase the biding affinity relative to DQ39 derivative, despite an increase in the charge density of its polymer chain. Further studies are needed to understand if a relative ‘stiffness’ of highly charged DQ56 polymer molecules in solution due to the effect of an increased number of ‘bulky’ pendant groups, and/or the ‘charge densities matching’ considerations contribute to this effect.

It should be noted that although non-quaternised derivatives, NQ30 and NQ60, possess no permanent positive quaternary charges on the piperazine ring, and hence could be expected to be significantly inferior at complexing siRNA than mono- and di-quaternary chitosans, the piperazine ring contains two tertiary amines that can be protonated in solution, in addition to ∼10% ionization of unsubstituted chitosan monomers at pH 7.4 (assuming pKa of 6.5) (Denuziere et al., 1996). The first pKa of 1,4-dimethylpiperazine (structure closely related to the substituent in NQ30 and NQ60) has been previously reported to be around 8.4 (Khalili et al., 2009), therefore at pH 7.4 ∼ 90% of the piperazine amine groups will be protonated, creating charge in NQ30 and NQ60. The second pKa was measured to be around pH 3.8, and hence this amine will not be appreciably protonated at pH 7.4 to contribute to the overall charge density of the polymers under experimental conditions used in the siRNA complexation. One also has to consider possible ‘local effects’ on ionization of piperazine amine groups in the vicinity of siRNA as a strong polyelectrolyte, as we observed previously (Ehtezazi et al., 2003).

Toxicity profiles of substituted chitosan derivatives (Supporting information, Figures S2 and S3) demonstrate no significant changes in cell metabolic activity up to 0.1 mg/ml. Two highly substituted mono-quaternary polymers, MQ1-85 and MQ4-87, have IC_50_ values around 0.9 mg/ml, and di-quaternary polymer with a high level of substitution, DQ56, has an IC_50_ around 0.05 mg/ml. For comparison, reported IC_50_ values for Lipofectamine and PEI are 0.05 mg/ml (Wen et al., 2004) and 6 µg/ml (Merkel et al., 2011), respectively, whilst application of a ‘non-modified’ chitosan with a comparable molecular weight (Ultrasan 20–70 applied at pH 6.0 due to chitosan’s low water solubility at physiological pH) caused a 73% reduction in viability at a concentration of 0.03 mg/ml (Casettari et al., 2010). Previous studies investigating the relationship between molecular weight (Mw) of chitosans, their cytotoxicity, and their siRNA binding affinity showed that ‘non-modified’ chitosans with MW <50 kDa, similar to molecular weight range of substituted chitosan used in this study, form siRNA complexes with larger particle size and poor transfection ability (Liu et al., 2007), or that those with MW <20 kDa did not form siRNA complexes (Ragelle et al., 2014). On the other hand, higher molecular weight chitosans, shown capable of siRNA complexation, have been linked to higher cytotoxicity (Huang et al., 2004). Despite their relatively low-molecular weight, the piperazine-chitosans used in the present study (∼MW 20–45 kDa) were capable of complexing siRNA at relatively low monomer:nucleotide ratios; improved binding can be attributed to the presence of the piperazine substituents (with permanent quaternary positive center in the MQ and DQ or two tertiary amines in NQ derivatives), which also improve water solubility while maintaining a relatively low cytotoxicity. It should be noted that ethidium bromide displacement assay showed that there was little benefit for the complexation of increasing the monomer:nucleotide ratio above 5:1, particularly in the case of the DQ39 derivative, with gel electrophoresis showing an appearance of excess of unbound, ‘free’ polymeric material above this ratio (Figure 1(B)). Therefore, in subsequent *in vitro* and *in vivo* studies, the monomer:nucleotide 5:1 ratio complexes were used. Data from particle size analysis illustrate the formation of nano-sized complexes of sub-300 nm diameter, for all piperazine-substituted chitosans at an optimized monomer:nucleotide ratio (Table S1), as well as a clear dependence of the particle size and complexes colloidal stability on monomer:nucleotide ratio (i.e. charge ratio, as per Table 1). DQ39-siRNA complexes possessed average zeta potential of +31 mV (Figure S4). Figure S5 illustrates that complexed siRNA can be released, with maintained integrity, from the DQ39 complexes.

**Figure 2.**
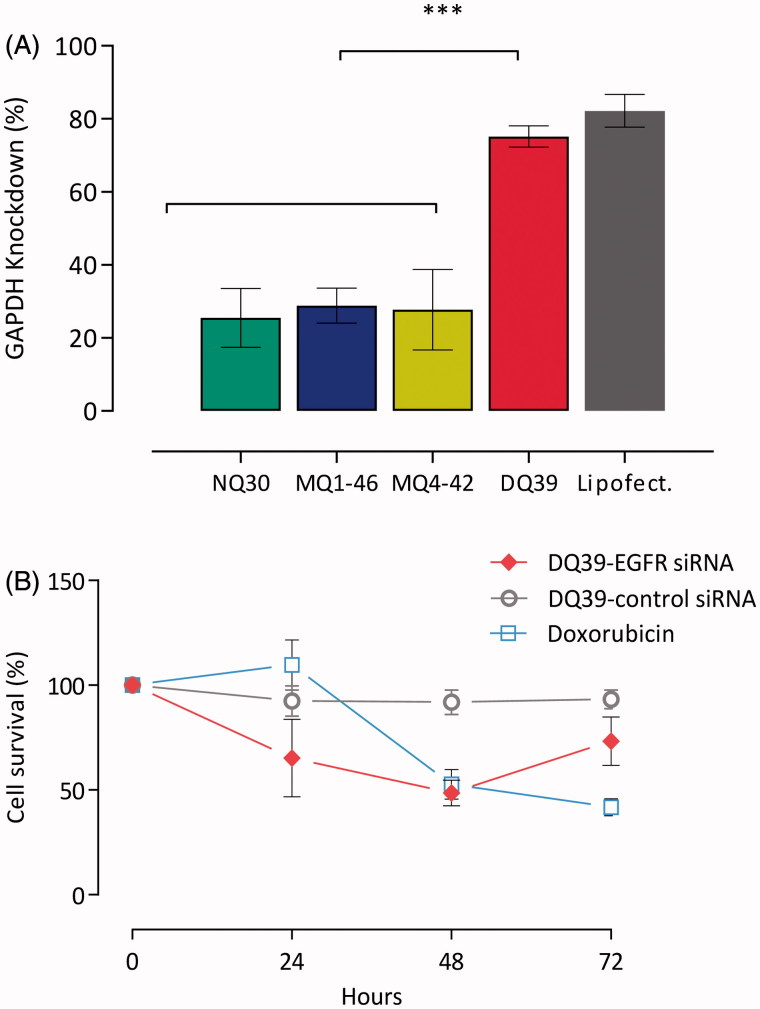
siRNA silencing with piperazine chitosan siRNA complexes. (A) Piperazine chitosan silencing of GAPDH in H1299 cells by siRNA complexes with different piperazine chitosan derivatives. Complexes (100 nM siRNA) were prepared at a 5:1 monomer:nucleotide ratio and incubated with cells in serum free RPMI-1640 medium for 4 hours and analysis performed 44 hours after complexes removal and cells incubation in fresh RPMI-1640 medium containing serum. GAPDH levels were evaluated using the KDalert GAPDH kit (Ambion) and expressed as the % GAPDH reduction relative to expression in untreated control cells. Data shown as the mean ± SD (*n* = 6). ***p < .001. (B) A549-*luc* cellular survival *in vitro*. A549-*luc* cells were seeded in a 96-well plate at a density of 10^4^ cells/well. EGFR-siRNA or negative control siRNA complexes with DQ39 (100 nM siRNA) were incubated with cells in serum-free medium for 4 hours. Samples were then removed and replaced with fresh serum-containing growth medium. Cells were incubated for a further 24, 48, or 72 hours after which cell survival was measured by an MTS assay. Doxorubicin (5 µM) was incubated with cells for 24, 48, or 72 hours. Doxorubicin was used at the IC50 concentration (5 µM) as established for 48 hr incubation in the previous experiment. Results are shown as the mean ± SD (*n* = 8).

It should be noticed that, with the exception of DQ56, all piperazine chitosans had an average charge density of less than 1 positive charge per monomer (Table 1), hence low levels of unbound charges would be present in the monomer:nucleotide 5:1 complexes used. On the contrary, the reports evaluating chitosan:siRNA complexes normally use high N:P ratios (N:*P* > 25, in some cases approaching 100:1) (Katas & Alpar, 2006). Such formulations could bring significant developability problems, including limited dosing and the potential toxic effects and burden of excess chitosan material. We hence propose that an additional comparative advantage of piperazine-substituted chitosans lays in their ability to form discrete, sub-300 nm sized complexes capable of silencing effect (Figure 2) at relatively low monomer:nucleotide ratios of ∼5:1. Although siRNA complexes with non- and mono-quaternary polymers, NQ30, MQ1-46, and MQ4-42, exhibited sub-30% gene silencing efficiencies (25%, 29%, and 28%, respectively), knockdown with the di-quaternary polymer, DQ39, reached ∼80% level, relative to untreated H1299 cells, and was comparable to lipofectamine (Figure 2). This gene silencing effect is substantial and compares favorably with that reported recently for trimethylated chitosan/tripolyphosphate nanoparticles incorporating siRNA (Ni et al., 2018). Analysis of silencing by siRNA:DQ39 polymer complexes in a panel of lung cell lines (A549, Calu-3, and H1299), in relation to the cell culture model and cellular trafficking, has been described previously (Capel et al., 2016). With the aim of the present study to investigate *in vivo* siRNA silencing potential of piperazine-chitosans, DQ39 complexes with EGFR-siRNA were evaluated in A549-*luc* transformed cells (Figure 2(B)), subsequently used to establish the bioluminescent tumor xenograft model in mice.

Data presented in Figure 2(B) indicate a time-dependent cell survival; at 24 and 48 hours post DQ39-(EGFR)-siRNA transfection, cell survival was reduced to 65% and 50%, respectively. At 72 hours post-transfection, it recovered to 70% of untreated cells, indicating the optimal treatment regime required for *in vivo* studies. Negative control siRNA had no significant effects on cell survival at any time-point (*p* > .05).

Developability potential of piperazine substituted soluble-chitosans as enabling technology for inhalation-mediated siRNA delivery was tested for nebulized formulation. Initially, to achieve osmolarity of physiological saline (0.300 osmol/kg) appropriate for inhalation, excipients (mannitol 4.6% *w/v*, sucrose 8.4% *w/v*, or trehalose 10.3% *w/v*) were included into the formation of DQ39-siRNA complexes and the aerolisation properties assessed using microspayer (PennCentury™) – a device utilized in intratracheal inhalation experiments in animals.

**Figure 3.**
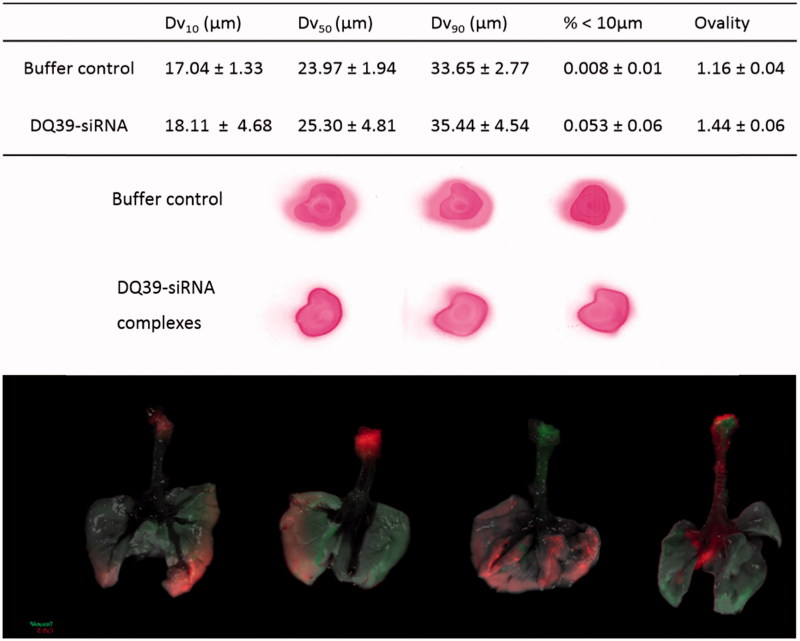
Typical droplet size analyses and spray pattern of DQ39-siRNA complexes sprays obtained by microsprayer (PennCentury™) and biodistribution of DY647-labeled DQ39-siRNA complexes in the lung following microsprayer administration to mice. Spray pattern analysis (top images) used Para Red dye added to the buffer for visualization purposes. Droplet size data (table) reported as volume diameter defined by 10%, 50%, and 90% (volume median) of the cumulative volume undersize (Dv10, Dv5, and Dv90, respectively). Data is given as the mean ± SD (*n* = 3). Ovality was calculated as the ratio of maximum vs. minimum diameter, average of three sprays, as illustrated. Bottom images illustrate IVIS analysis of excised lung following application by a microsprayer of DY647-labeled siRNA complies with DQ39 formulated at 5:1 ratio at siRNA concentration of 10 µg/50 µl. Mice were dosed with siRNA complexes (10 µg siRNA in 50 µl) and images of obtained by IVIS imaging immediately after administration.

Data from *in vivo* animal studies following administration of the DQ39-siRNA complexes as aerosol via microsprayer (PennCentury) in mice are illustrated in Figure 3. Aerosols produced by this microsprayer have been demonstrated to reach the lung periphery, in spite of the relatively large droplet size (similar as in Figure 3), and effectively preserve the integrity and viability of biological materials (Bivas-Benita et al., 2005). In a previous work, deposition siRNA complexes following aerolisation by a microsprayer was assessed in alveolar and bronchiolar tissue sections of the lung (Nielsen et al., 2010), however, IVIS analysis used in this study provided a valuable information of the whole lung deposition pattern. The data illustrate that siRNA distribution throughout the lung does not appear homogeneous and that the deposition pattern varies within the lung and between animals (Figure 3). Unfortunately, following lung administration, we were not able to detect the DY647-siRNA-DQ39 fluorescence in the lung in whole, intact animals using IVIS imaging. We hence imaged excised lungs for the deposition studies. The possible reason may be low penetration of fluorescence across coastal bones structures, despite a far-red emitter, DY647 probe. This furthermore prevented the use of IVIS to assess *in vivo* silencing of the complexes in an orthotopic lung tumor model.

**Figure 4.**
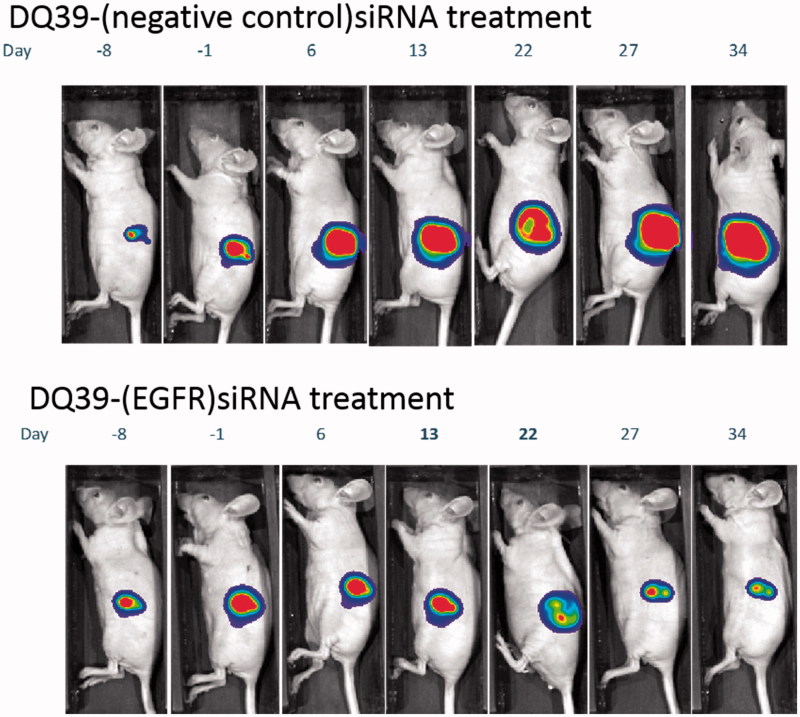
Tumour growth inhibition by EGFR-siRNA complexes with DQ39 piperazine-substituted chitosan. Images show luciferase bioluminscence of A549*Luc* cells assessed by whole animal bioluminescent imaging (IVIS) of DQ39-(negative control)-siRNA (upper images) and DQ39-(EGFR)-siRNA complexes (lower images) at day −8, −1, 6, 13, 22, 27, and 34 of the treatment. IVIS images following one selected mouse per group are shown. Graph shows mean bioluminescent intensity relative to pretreatment values.

*In vivo* potential of piperazine chitosan complexes to inhibit tumor growth through the silencing of EGFR was therefore assessed in subcutaneous bioluminescent tumor A540-*luc* xenograft model. Mice were treated twice weekly *via* intratumoural injection with either DQ39-(EGFR)-siRNA or DQ39-(negative control)-siRNA complexes for four weeks. The effects of the treatments on tumor growth were monitored by whole body *in vivo* bioluminescent imaging (IVIS). Data are presented in terms of mean tumor bioluminescent intensity (BLI) and typical IVIS images from one animal representative of each treatment group at each time point throughout the study (Figure 4). Prior to the therapy study, acute tolerability of the siRNA, the delivery system, and doxorubicin standard of care was assessed in tumor bearing mice (two mice per treatment under dosing as per the therapy study for 14 days, followed by cessation of dosing and observation for a further 14 days). No adverse effects were noted in the tolerability study and subsequent therapy study. Adverse effects (diarrhea, weakness, and severe weight loss) were described in a previous study, which reported successful inhibition of tumor growth on the treatment with EGFR and VEGFR targeted siRNA complexed by PEI (Chen et al., 2013). In our therapy study, administering DQ39-(EGFR)-siRNA complexes, no such adverse effects were observed over repeat dosing for four weeks.

**Figure 5.**
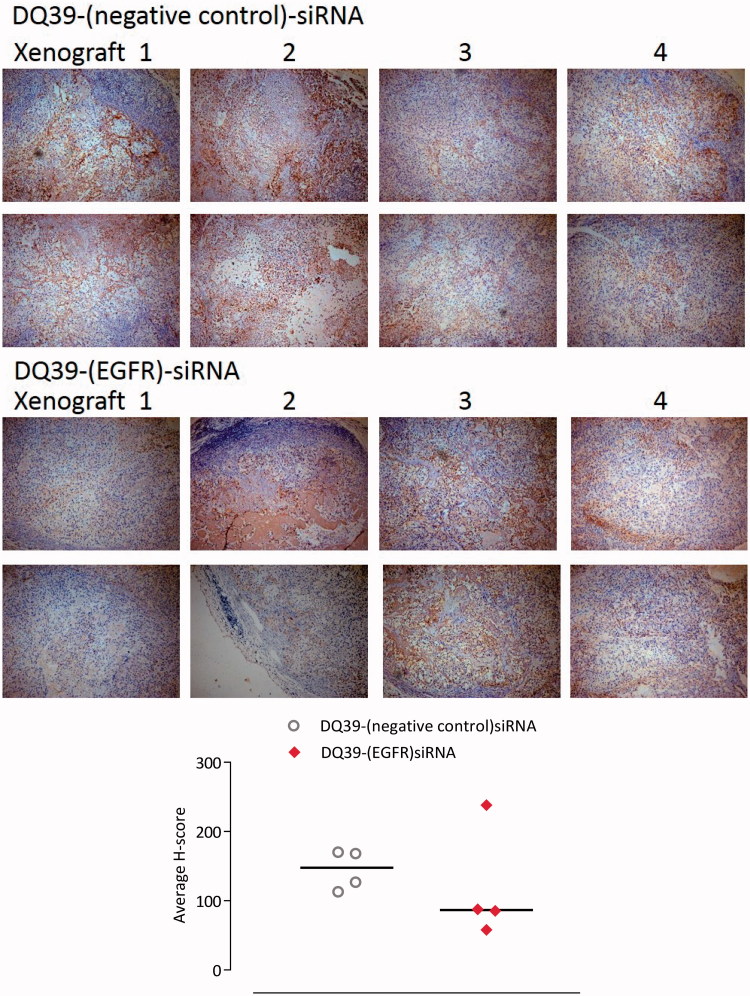
Mouse xenograft models stained for EGFR. Samples of A549*Luc* xenograft mouse models excised at the final time point. Four xenograft samples from each treatment group were stained for EGFR (two images of different fields of view shown for each xenograft). Images taken with a Leica DM4000B light microscope at ×10 magnification. Areas expressing EGFR are stained brown (darker regions in black and white copy). Graph shows H-scoring data, as explained in the experimental section.

The data illustrate that EGFR-siRNA complexes with DQ39 piperazine-substituted chitosan were able to inhibit tumor growth, relative to negative control siRNA treatment. Further analysis of the complexes’ silencing effect was conducted by histological inspection of tumor xenografts extracted at the final treatment time point (Figure 5). The data show that administration of EGFR-siRNA complexes significantly inhibited EGFR protein level, and support evidence from previous work (Chen et al., 2013), although the reasons for maximum silencing effect occurring at 20 days of the treatments are not clear at present. It should be noted that the xenografts were excised at the final time point, following day 34 of treatment, and that hence they do not represent the point in the treatment at approximately day 20 when the EGFR level reduction was the most significant (Figure 5). It is therefore important to notice that the EGFR levels were still significantly reduced in tissue samples of animals treated with the EGFR siRNA complexes, relative to negative control.

## Conclusions

Piperazine substituted chitosans are, unlike unmodified chitosan, freely soluble in water at physiological pH. They afford an efficient complexation of siRNA into sub-300 nm colloidal complexes, achieved at relatively low polymer to siRNA monomer: nucleotide ratio of 5:1 for a DQ39 di-quaternary derivative. Substituted chitosans possess low cytotoxicity, with no significant reduction in cell viability up to 0.1 mg/ml concentration , in comparison to 0.03 mg/ml for pure, lower molecular weight chitosan (when applied dissolved at pH 6.0, due to solubility issues). *In vitro*, the complexes ensure significant silencing of ∼80% for best performing DQ39-siRNA complexes. *In vivo*, the piperazine chitosan complexes did not cause tolerability issues and achieve successful inhibition of tumor growth in subcutaneous bioluminescent tumor A540-*luc* xenograft model. Developability of the system as aerosol formulation for inhalation delivery is also demonstrated, illustrating a potential of modified chitosans to be utilized as a safe system for delivery of siRNA.

## Supplementary Material

IDRD_Stolnik_et_al_Supplemental_Content.docx
