# Supplementary material for: Water-soluble substituted chitosan derivatives as technology platform for inhalation delivery of siRNA
Source: Drug Deliv. 2018 Mar 1;25(1):644–53. doi: 10.1080/10717544.2018.1440668 (PMC6058492; doi:10.1080/10717544.2018.1440668)
Supplement: IDRD_Stolnik_et_al_Supplemental_Content.docx [file IDRD_A_1440668_SM3139.docx]

**Supporting Information**

**Water-soluble substituted chitosan derivatives as technology platform for inhalation delivery of siRNA**

Victoria Capel, Driton Vllasaliu, Peter Watts, Philip A Clarke, Dominic Luxton, Anna M Grabowska, Giuseppe Mantovani and Snjezana Stolnik


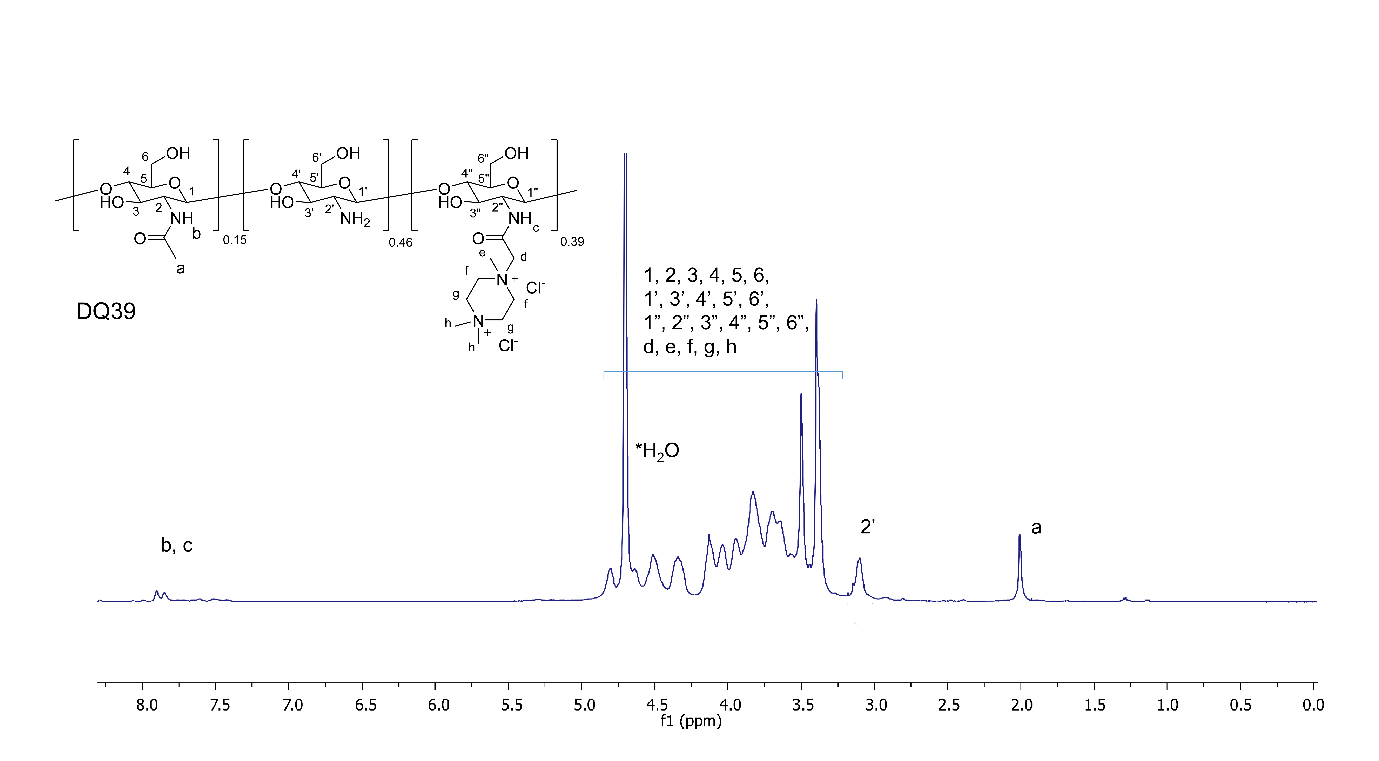


**Figure S1. Representative ^1^H NMR spectrum of a piperazine chitosan polymers**. Spectrum of ^1^H NMR of DQ39 is shown. The degree of quaternisation of the commercially available polymers was confirmed by comparing the integral of the signal of the C_2’_*H*-NH_2_ protons at 3.1 ppm, to that of the NHC(O)C*H*_3_ at 2.0 ppm (chitosans used in this work had 15% residual acetylated amino groups (Holappa et al., 2006)).

Figure S2. Cytotoxicity of piperazine chitosans in H1299 cells. Polymers were applied in serum-free medium for four hours, after which cellular viability was measured by an MTS assay. Data shown as mean +/- SD (n = 8).

Figure S3. Cytotoxicity of DQ39 in epithelial cell lines. Cells were incubated with DQ39 at a concentration range of 0.0001 to 1 mg/ml for four hours. Cell metabolic activity was measured using an MTS assay and compared to media (100% viability) and Triton X (0% viability). Data shown as the mean +/- SD (n=8).

| Monomer:  nucleotide ratio | Mean diameter (SD) / nm | | | | | | | |
| --- | --- | --- | --- | --- | --- | --- | --- | --- |
|  | MQ1-46 | MQ1-85 | MQ4-42 | MQ4-87 | DQ39 | DQ56 | NQ30 | NQ60 |
| 0.5:1 | 121.2 (14.2) | ** | 233.9 (21.5) | ** | 255.9 (10.8) | 266.5 (32.2) | ** | ** |
| 1.0:1 | 272.1 (16.0) | 313.4 (16.0) | 476.2 (18.9) | 203.0 (26.0) | 252.5 (17.5) | 298.0 (18.6) | 225.7 (35.1) | 274.3 (20.9) |
| 2.0:1 | 1008.3 (70.5) | 595.3 (92.0) | 888.5 (104.8) | 1026.0 (205.1) | 234.3 (6.2) | 168.0 (18.9) | 293.3 (16.2) | 1076.4 (129.3) |
| 5.0:1 | 248.3 (20.4) | 336.9 (51.3) | 210.9 (23.4) | 251.8 (24.8) | 135.7 (15.0) | 150.3 (5.8) | 955.7 (16.7) | 287.9 (24.2) |
| 10.01 | 219.9 (11.7) | 253.6 (41.7) | 180.9 (2.8) | 202.0 (9.3) | 125.3 (8.2) | 126.9 (5.6) | 211.7 (19.1) | 327.3 (20.8) |

Table S1. Mean hydrodynamic particle size diameter, determined by dynamic light scattering, of piperazine chitosan-siRNA complexes.

Complexes were formed in 10 mM Tris-HCl buffer (pH 7.4); results are given as the mean (SD) of three independent measurements (each measurement average of 10 runs) at 25°C, as measured by dynamic light scattering. ** Insufficient scattering to conduct measurements.


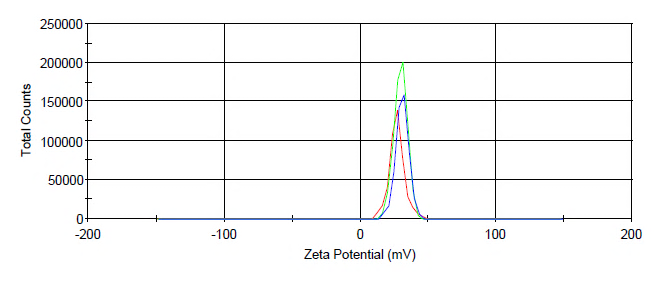


**Figure S4. Typical zeta potential measurements profile of siRNA-polymer complexes**. siRNA-DQ39 complexes prepared at ratio 5:1, with average zeta potential of +31mV.

**
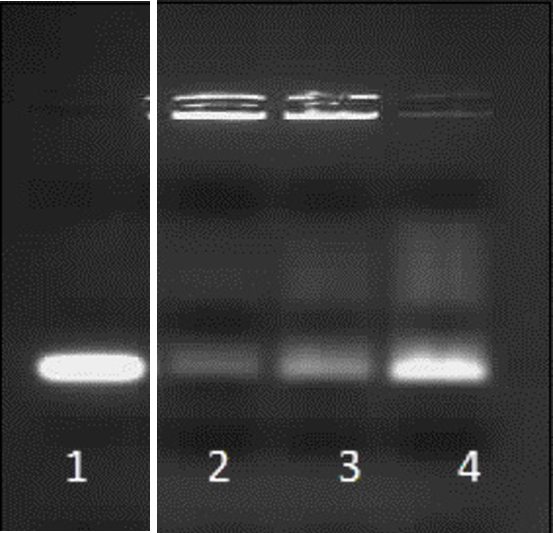
**

**Figure S5. Release of siRNA in a ‘competitive’ environment**. siRNA complexes based on DQ39 were incubated with heparin and released siRNA assessed by gel electrophoresis. Lane 1: siRNA control; lanes 2-4: siRNA-DQ39 complexes plus heparin (0, 0.1, 0.2 U/µg siRNA, respectively).

**References**

Holappa, J., Nevalainen, T., Safin, R., Soininen, P., Asplund, T., Luttikhedde, T., Masson, M., Jarvinen, T. (2006). Novel water-soluble quaternary piperazine derivatives of chitosan: synthesis and characterization. Macromol Biosci 6, 139-144.
